# Intraoperative quantification of fluorescence angiography for assessment of intestinal perfusion: *in vivo* exploration of clinical value

**DOI:** 10.1093/bjsopen/zrac058

**Published:** 2022-05-06

**Authors:** Harry G. M. Vaassen, Bryan Wermelink, Srirang Manohar, Robert H. Geelkerken, Daan J. Lips

**Affiliations:** 1 Multi-Modality Medical Imaging (M3I) group, TechMed Centre, University of Twente, Enschede, The Netherlands; 2 Section Vascular Surgery, Department of Surgery, Medisch Spectrum Twente, Enschede, The Netherlands; 3 Section Gastrointestinal and Oncology Surgery, Department of Surgery, Medisch Spectrum Twente, Enschede, The Netherlands; 4 Dutch Expert Centre for Gastrointestinal Ischaemia, Medisch Spectrum Twente, Enschede, The Netherlands

*Dear Editor*

Perfusion status of the intestine is regarded as one of the most critical factors for anastomotic leakage specifically, and intestinal viability in general^1^. Currently this is evaluated by means of clinical observations such as serosal discoloration and arterial pulsation; however, this is subjective, and it has been demonstrated that surgeons lack predictive accuracy regarding AL^2^. The decision to resect suspected non-viable tissue in the case of acute mesenteric ischaemia or bowel incarceration is often based on clinical inspection and surgeon’s experience. The surgeon must balance the risk subjectively of leaving non-viable bowel *in situ versus* small bowel resection and consequent risk of short bowel syndrome. There is a need for objective and reliable intraoperative assessment of intestinal perfusion.

Fluorescence angiography (FA) with indocyanine green (ICG) provides valuable insights into local perfusion dynamics, but qualitative interpretation of images can be deceptive and seems to be inadequate in reducing AL^3^. Quantitative assessment may improve interpretation but remains challenging due to the lack of standardization, absence of robust metrics for ICG kinetics, and paucity of reference quantified data in healthy and impaired perfusion situations^4^.

This study presents the first clinical findings after implementing a method that allows semi-automatic live quantification of bowel perfusion based on FA inflow parameters.

Algorithms for the onsite extraction of FA inflow parameters time to peak (TTP) and normalized peak slope (NPS) were developed (*Appendix S1*). An accompanying in-house-built software tool enabled real-time quantified analysis and integration of parametric mapping with live video footage.

A database of 32 FA recordings in patients without impaired bowel perfusion was retrospectively analysed to provide a reference of ‘healthy’ normal values^5^. Nine patients with suspected bowel perfusion impairment, of which seven underwent a restoration of vascularization, were prospectively included. These procedures consisted of five open superior mesenteric artery reconstructions for chronic mesenteric ischaemia, two laparotomies for acute mesenteric ischaemia, and two laparoscopic reductions of small bowel strangulation. Characteristics of these patients, diagnoses, and performed interventions are presented in *Table S1*. FA was performed on small bowel segments after intravenous administration of 5 mg ICG, before, and after revascularization.

Analysis of the group with unimpaired bowel perfusion provided a median (interquartile range (i.q.r.)) TTP of 4.8 (2.5) s, with 95 per cent of measurements under 7.0 s. Median (i.q.r.) NPS was found to be 12.8 (5.1) per cent/s, with 95 per cent of measurements more than 9.4 per cent/s.

The group with suspected impaired perfusion showed a median (i.q.r.) TTP of 9.6 (3.2) s and median (i.q.r.) NPS of 7.3 (1.9) per cent/s, both significantly different compared with the unimpaired group (*P* = 0.002). Measurements after vascularization was restored demonstrated a median (i.q.r.) TTP of 4.2 (1.6) s and a median (i.q.r.) NPS of 14.5 (3.3) per cent/s, which differed significantly from the median before intervention (*P* = 0.033). Seven out of nine patients with suspected impaired bowel perfusion displayed inflow parameters outside the healthy range. Six of the seven patients who underwent revascularization showed evident improvement of inflow parameters, as well as resolution of symptoms (see example in *Fig. 1*). The remaining patient suffered from recurrent atypical abdominal symptoms. All measurements are presented in *Table S2* and *Fig. S1*.

**Fig. 1:**
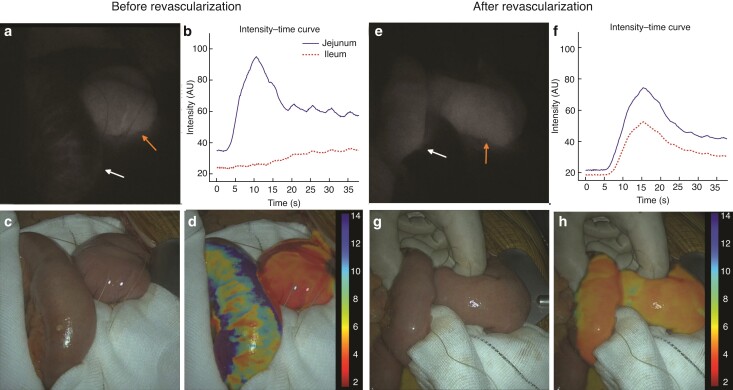
**Intraoperative fluorescence angiography analysis of a patient with chronic mesenteric ischaemia (Pt. A4) before (left) and after (right) superior mesenteric artery revascularization**. Left arrow indicates ileum, and right arrow indicates jejunum. **a**,**e** Monochromatic near-infrared imaging mode. **b**,**f** Intensity-over-time curves of the jejunal and ileal segment. AU, arbitrary units. **c**,**g** White-light imaging mode. **d**,**h** Heat map based on time to peak overlayed on white-light footage.

This study suggests that this method for onsite FA quantification yields inflow-based parameters that enable discrimination between healthy and impaired perfusion and reflect corresponding symptoms. Further sufficiently powered clinical trials are being conducted to define FA thresholds for various perfusion requirements.

*Disclosure*. The authors declare no conflict of interest.

## Supplementary material

Supplementary material is available at *BJS Open* online.

## Supplementary Material

zrac058_Supplementary_Data
