# Supplementary material for: Intraoperative quantification of fluorescence angiography for assessment of intestinal perfusion: in vivo exploration of clinical value
Source: BJS Open. 2022 May 6;6(3):zrac058. doi: 10.1093/bjsopen/zrac058 (PMC9072211; doi:10.1093/bjsopen/zrac058)
Supplement: zrac058_Supplementary_Data [file zrac058_supplementary_data.zip › Supplementary_material.docx]

***Intra-operative Quantification of Fluorescence Angiography for Assessment of Intestinal Perfusion: An In Vivo Exploration of Clinical Value***

Supplementary Material

## Appendix S1: Methods of analysis

Fluorescence angiography data was analysed using an in-house developed software tool, written in Python (Python Software Foundation, Wilmington, DE, USA). RGB frames were captured at 10 frames/second and converted to a luminance map. Intensity-over-time curves were generated by monitoring the luminance value of a specific pixel or region of interest (ROI) along the data frames. Two parameters were defined for analysis: time to peak (TTP) and normalized peak slope (NPS). TTP was defined as the time between reaching I_20%_ and I_80%_, which are calculated according to:

$$I_{20\%}=I_{0\%}+0.2\times\left( I_{100\%}-I_{0\%} \right)$$

$$I_{80\%}=I_{0\%}+0.8\times\left( I_{100\%}-I_{0\%} \right)$$

I_0%_ and I_100%_ are the baseline and maximum intensity, which are calculated by taking the median value of respectively the ten lowest and ten highest data samples of the curve.
For determining NPS, the signal is convolved with a finite impulse response differentiation filter with a window size of 75 data points. This operation filters the signal and computes the first derivative. Peak slope (PS) is the maximum value of the output and is considered as the maximal slope in the signal that is not originating from high frequency noise or artefacts such as peristalsis. For the purpose of being solely inflow-based, PS is normalized and converted to units [%/second] by:

$$NPS=\frac{PS}{I_{100\%}-I_{0\%}}\times10\times100\%$$

After indocyanine green inflow has occurred, the software immediately allows the user to interactively draw and move ROI’s over either images from the FA recording or the live video footage, enabling direct signal quantification and automated analysis of the desired bowel segment. Moreover, both parameters could be calculated for all pixels individually and displayed as a heatmap within 5-10 seconds, providing instant integration of the quantification in the live intraoperative (white light) footage.

|  | | | | | | | | |
| --- | --- | --- | --- | --- | --- | --- | --- | --- |
| **Table S1.** Baseline characteristics and specifics on diagnosis and performed intervention of patients with suspected bowel perfusion impairment (*n* = 9). | | | | | | | | |
| **Group** | **Pt. #** | **Sex** | **Age** | **BMI** | **ASA** | **Vascular risk factors** | **Diagnosis** | **Intervention** |
| A | 1 | F | 78 | 24.2 | IV | - | 2-Vessel (CA/SMA) CMI | CIA to SMA graft* |
|  | 2 | F | 53 | 35.9 | II | HT, HL | 2-Vessel (CA/SMA) CMI | EIA to SMA graft^#^ |
|  | 3 | M | 66 | 29.3 | IV | DM2, HT, HL, PAD, Smoking | 3-Vessel AoCMI | Antegrade aortic to SMA graft* |
|  | 4 | M | 56 | 24.8 | III | - | 1-Vessel (SMA) AoCMI | Patch angioplasty SMA^#^ |
|  | 5 | F | 38 | 22.7 | III | PAD, Smoking | 3-Vessel CMI | Aortic endarterectomy, retrograde aortic to SMA and CHA graft^#^ |
| B | 1 | M | 57 | Unknown | V | DVT, PAD | 3-Vessel AMI | Laparotomy – no further actions |
|  | 2 | M | 65 | 21.2 | IV | CVA, HT, PAD | 2-Vessel AoCMI | Ileum/coecum resection |
| C | 1 | M | 63 | 24.1 | I | Smoking | Herniation-induced SBS | Laparoscopic release |
|  | 2 | M | 61 | 27.2 | II | AF, HT, HL, Smoking | Adhesion-induced SBS | Laparoscopic release |
| *AF,* atrial fibrillation; *AMI,* acute mesenteric ischemia; *AoCMI,* acute-on-chronic mesenteric ischemia; *ASA,* American Society of Anaesthesiologists; *BMI,* body-mass index; *CA,* coeliac artery; *CHA,* common hepatic artery; *CIA,* common iliac artery; *CMI,* chronic mesenteric ischemia; *CVA,* cerebrovascular accident; *DM2,* type 2 diabetes mellitus; *DVT,* deep vein thrombosis; *EIA,* external iliac artery; *HL,* hyperlipidaemia; *HT,* hypertension; *PAD,* peripheral artery disease; *SBS,* small bowel strangulation; *SMA,* superior mesenteric artery; * Prosthetic; ^#^ Autogenous | | | | | | | | |

| **Table S2.** Results of quantified FA analysis in patients with impaired mesenteric circulation. | | | | | | | | | |
| --- | --- | --- | --- | --- | --- | --- | --- | --- | --- |
| **Group** | **Pt. #** | **TTP (seconds)** | | |  | **NPS (%/second)** | | | **Clinical judgement*** |
|  |  | **Pre** | **Post** | **Reference** |  | **Pre** | **Post** | **Reference** |  |
| A | 1 | 8.7 | 4.7 | - |  | 7.8 | 15.0 | - | VI – PI |
|  | 2 | 5.8 | 6.4 | - |  | 10.8 | 11.7 | - | VI – PI |
|  | 3 | 10.2 | 3.9 | - |  | 6.6 | 18.5 | - | VI – PI |
|  | 4 | 18.6 | 4.2 | - |  | 6.2 | 14.5 | - | VI – PI |
|  | 5 | 10.9 | 8.5 | - |  | 6.1 | 8.6 | - | VI – PI |
| B | 1 | 8.9 | - | - |  | 7.5 | - | - | VI – PII |
|  | 2 | 8.2 | - | - |  | 8.6 | - | - | VII – PI |
| C | 1 | 11.8 | 4.1 | 3.7 |  | 7.1 | 14.2 | 15.2 | VIII |
|  | 2 | 6.9 | 3.9 | 4.3 |  | 9.1 | 17.5 | 15.5 | VII |
| Control (median) | | 4.8 (IQR = 2.5) | | |  | 12.8 (IQR = 5.1) | | | VI |
| ***** Scored before revascularization based on visual inspection (V) and palpation (P) as either I (most likely reversible or no transmural ischemia), II (dubious reversibility of ischemia) or III (most likely irreversible, transmural ischemia/necrosis)  **Data availability**  Analytic methods are part of ongoing software and analytical development and are confidential, with exception of those mentioned in Appendix S1. As of now, software, and analytics cannot be made available. Intraoperative fluorescence imaging videos will not be made publicly available. This research was not preregistered with an analysis plan in an independent, institutional registry. Data and other artefacts have not been archived in a public repository. | | | | | | | | | |
