# Supplementary figures and images for: Intraoperative quantification of fluorescence angiography for assessment of intestinal perfusion: in vivo exploration of clinical value
Source: BJS Open. 2022 May 6;6(3):zrac058. doi: 10.1093/bjsopen/zrac058 (PMC9072211; doi:10.1093/bjsopen/zrac058)

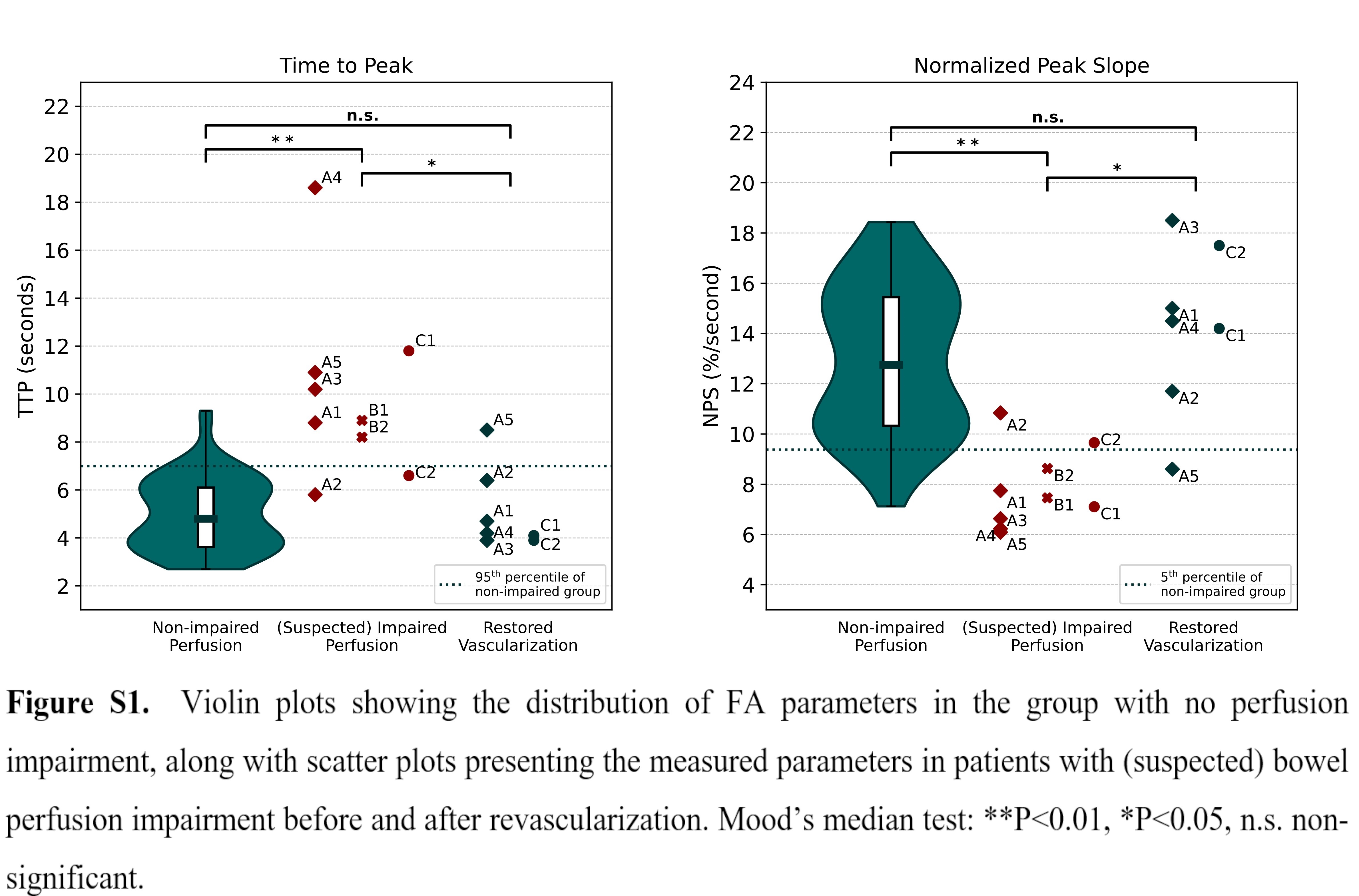

Supplement: zrac058_Supplementary_Data [file zrac058_supplementary_data.zip › Supplementary_Figure_1.jpg]
